# Characterization of human malignant mesothelioma cell lines orthotopically implanted in the pleural cavity of immunodeficient mice for their ability to grow and form metastasis

**DOI:** 10.1186/1471-2407-6-130

**Published:** 2006-05-17

**Authors:** Daniele Martarelli, Alfonso Catalano, Antonio Procopio, Sara Orecchia, Roberta Libener, Giorgio Santoni

**Affiliations:** 1 Department of Experimental Medicine and Public Health, University of Camerino, 62032 Camerino, Italy; 2 Department of Molecular Pathology and Innovative Therapies, Polytechnic University of Marche, 60100, Ancona, Italy and Center of Cytology, Italian National Research Centers on Aging (INRCA – IRCCS), Ancona, Italy; 3 Pathology Unit, Dept. Of Oncology, A.S.O. Alessandria, Italy

## Abstract

**Background:**

Malignant pleural mesothelioma (MPM) is a tumor known to be resistant to conventional therapies. Thus, an in vivo model can represent an important tool for assessing the efficacy of novel approaches in the treatment of MPM.

Presently, human MPM cells have been grown orthotopically in mice upon transplantation of tumor masses or tumor cell suspensions following surgery. In these models however, surgery can interfere with the tumor growth and the early stages of tumor development cannot be easily explored. Finally, results may not be so accurate due to implantation of potentially different tumor samples in different experimental groups.

Our work aimed at establishing a nude mouse model xenotransplanted with human MPM cell lines in which tumor progression exhibits some features of the human disease.

**Methods:**

Three distinct human MPM cell lines previously established from MPM patients displaying two different phenotypes, biphasic (MM-B1 and IST-Mes3) and epithelioid (IST-Mes2), were directly injected into the pleural cavity of nude mice. At different times, mice were sacrificed for autopsy, tumor nodules were counted and then removed for histology. Presence of metastases in visceral organs was also monitored.

**Results:**

IST-Mes2 cells were unable to grow in nude mice. MM-B1 and IST-Mes3 cells were capable of growing in nude mice and formed tumor nodules in the pleura. Post-mortem examination showed that MPM cells progressively colonized the parietal and visceral pleura, the diaphragm, the mediastinum and, lastly the lung parenchyma. No pneumo-thorax was evidenced in the mice. Pleural effusions as well as lymph node metastases were observed only at later times.

**Conclusion:**

This model mimics the progression of human malignant mesothelioma and it is easy to perform and reproducible; therefore it can be useful to study human MPM biology and evaluate the efficacy of novel therapies.

## Background

Malignant pleural mesothelioma is a tumor of the pleura mainly caused by exposure to asbestos fibers. MPM diagnosis regards about 2500 persons every year in the United States [1] and the prognosis is poor despite the therapies currently used, including surgery, radiotherapy and chemotherapy.

Because of the inefficacy of the conventional treatments, novel therapeutic strategies are under investigation, with particular attention devoted to agents capable of inhibiting the angiogenesis or inducing tumor cell apoptosis [2-7].

Tumor angiogenesis, apoptosis and metastasis, strictly depend on the site of tumor development; therefore a human-like animal model is an important tool for studying new approaches for MPM treatment.

A number of evidences indicate that orthotopic models of tumor growth are more valuable as compared to those in which the tumor mass grows subcutaneously [8].

In regard to MPM, subcutaneous implantation of human cancer cells in immunodeficient mice results in tumor growth at the injection site and no metastatic dissemination, whereas human MPM growth in humans is associated with regional tumor spreading and lung invasion [9].

Presently, human MPM cells have been grow orthotopically in mice upon transplantation of tumor masses or tumor cell suspensions following surgery [10]. In these models however, surgery can interfere with the tumor growth [8] and the early stages of tumor development cannot be easily explored. Finally, results may be not so accurate due to implantation of potentially different tumor samples in different experimental groups [9]. Overall, the aim of this work was to establish a new orthotopic tumor model by injecting human MPM cells directly into the pleural cavity of nude mice. This model does not need surgical operations, can be easily performed and more importantly can mimic tumor development in humans. Thus it can represent an useful tool for studying human MPM biology and assessing the efficacy of novel therapies.

## Methods

### Cell lines

Human malignant pleura mesothelial cell lines were established as previously described [11,12]. Three distinct cell lines with two different phenotypes, biphasic (MM-B1 and IST-Mes3) and epithelioid (IST-Mes2) were used between the eighth and twelfth passage in culture. Cells were maintained in RPMI-1640 medium and 10% fetal bovine serum, 1% L-glutamine and 1% penicillin-streptomycin, (Euroclone, Devon, UK) at 37°C in a humidified incubator in an atmosphere of 5% CO2 in air.

### Animals

Athymic male nude mice nu/nu (Harlan, Italy), 6 week-old were used. Mice were kept in laminar-flow cages in standardized environmental conditions. Sterilized food (Harlan, Italy) and water were supplied ad libitum.

### Subcutaneous implantation of MPM cells in nude mice

Cells were harvested at near confluence with trypsin/EDTA solution. Only cell suspensions with a viability of >90% as assessed by trypan blue exclusion assay, were used. Two × 10^6 ^MPM cells in 100 μl of Ca++ and Mg++ free Hank's balanced salt solution (HBSS) were injected subcutaneously on the left lateral chest wall near to the axilla. Tumor growth was monitored twice a week using a caliper. Tumor volume was calculated using the formula: V (mm3) = (D × d2)/2, where d (mm) and D (mm) are the smallest and largest perpendicular tumor diameter, respectively.

### Orthotopic implantation of MPM cells in nude mice

Mice were anaesthetized with Tiletamine chlorohydrate and Zolazepam chlorohydrate and placed in position of right lateral decubitus. A 27 gauge needle of a 1000 μl syringe was advanced approximately through the fourth intercostal space for about 5 mm, into the left pleural cavity, and two × 10^6 ^tumor cells suspended in 100 μl of HBSS were injected. The site of injection in the chest and the precise depth of the needle tip required to reach the pleural space were previously determined by injecting cresyl violet. A primary tumor cell line IST-Mes3/2P (IST-Mes3/2nd Passage) was established from a IST-Mes3 tumor grown orthotopically. Tumors were grounded into small pieces in RPMI-1640 and digested with trypsin/EDTA solution. The cell suspension was then plated in a 75 ml culture flask and the following day, non adherent cells were removed. Cells were maintained in culture, and 2 × 10^6 ^cells were injected into the pleural cavity of nude mice when devoid of fibroblasts or endothelial cells.

### Therapeutic procedures

Four groups of animals were used. The first group of animals were used to analyze the tumorigenicity of MPM cell lines after orthotopic or subcutaneous implantation in nude mice. Animals were then injected with MPM cells as reported in Table 1.

The second, third and fourth groups were used to analyze the growth rate of IST-Mes3, IST-Mes3/2P and MM-B1 cells respectively after orthotopic implantation in mice.

### Autopsy and histology

Mice were sacrificed at different times following tumor cell injection as specified.

Tumor nodules were counted, measured with the caliper, removed, immediately snap frozen in liquid nitrogen and stored at -80°C. The presence of metastasis in the visceral organs was macroscopically checked.

### Immunohistochemistry

Microvessel density (MVD) was determined by using the endothelial cell marker CD31. Tumors were placed in OTC compound and snap frozen in liquid nitrogen and stored at -80°C. Frozen section (10–20 μm) were fixed with cold acetone (5 min), acetone/chlorophorm 1/1 (5 min) and cold acetone (5 min). Samples were then rinsed with PBS/Triton 1%, and treated with 3% hydrogen peroxide in methanol (vol/vol). Slides were incubated in a blocking solution and then overnight at 4°C in a humidified chamber with a rat anti-mouse CD31 monoclonal antibody (BD Biosciences Pharmingen, NJ, USA). Thereafter, slides were rinsed with PBS and incubated, first with the blocking solution for 20 min and then with a biotin-conjugated goat anti-rat antibody (Santa Cruz Biotechnology, CA, USA) for 1 h. Slides were then rinsed with PBS and incubated for 30 min with the Vector Vectastain ABC Kit (Vinci-Biochem, Vinci, FI). After 3 washes with PBS, positive reactions were visualized by incubating the slides for 5 min with stable DAB (Sigma, Italy). Slides were dried and mounted with Universal Mount.

For mesothelioma markers, 3–4 μm paraffin tumor sections were float mounted on poly-L lysine coating slides, deparaffinized in xylene and rehydrated in a descending ethanol series. Anti-Ber-EP4 monoclonal antibody required enzymatic pre-treatment of the slides with 0.1% trypsin (Sigma, St. Louis, MO) for 10 min at 37°C; for the other antibodies, slides were not enzimatically pre-treated, but they were placed in 0.1 M citrate buffer (pH 6.0) and boiled for 10 min in a microwave oven at 750 W to enhance antigenicity. Slides were washed in phosphate buffer and incubated for 10 min in 0.3% hydrogen peroxide to quench endogenous peroxidase activity. Slides were then loaded onto a LabVision automated immunostainer (NeoMarkers, Fremont, CA) and sequentially incubated with intervening washes in PBS for 5 min, with 10% ovalbumin for 15 min to reduce non-specific background staining, primary antibody for 60 min at room temperature, the appropriate biotinylated linking antibody (NeoMarkers, Fremont, CA) for 10 min, peroxidase-labeled streptavidin (NeoMarkers, Fremont, CA) for 10 min, and finally with 3,3'-diaminobenzidine chromogen substrate for 10 min. Slides were then thoroughly rinsed in distilled water and counterstained with Mayer's haematoxylin, dehydrated, cleared in xylene and finally mounted in Entellan. Appropriate positive and negative controls were included for each marker. A panel of eight markers was used. Mouse anti-human antibodies included anti-CEA (dilution 1:100; NeoMarkers, Fremont, CA), polyclonal anti-calretinin (dilution 1:1000; NeoMarkers, Fremont, CA), monoclonal anti-CD15 (Leu-M1) (dilution 1:4; Becton Dickinson, NJ), monoclonal anti-Ber-EP4 (dilution 1:100; NeoMarkers, Fremont, CA), monoclonal anti-cytokeratin 8 and 18 (dilution 1:50; Biomeda, Foster City, CA), monoclonal anti-EMA (dilution 1:500; NeoMarkers, Fremont, CA). monoclonal anti-mesothelioma (HBME-1) (dilution 1:25; NeoMarkers, Fremont, CA), monoclonal anti-podoplanin (dilution 1:50; Serotec, Dusseldorf, Germany).

### Hematoxylin-and-eosin staining

Tumors were placed in OTC compound and snap frozen in liquid nitrogen and stored at -80°C. Frozen section (10–20 μm) were fixed with cold acetone (5 min), acetone/chlorophorm 1/1 (5 min), cold acetone (5 min). Slides were then rinsed with water for10 min, stained with Mayer's Hematoxylin Solution for 10 min (Sigma, St. Louis, MO), rinsed with water for 10 min and then stained with eosin 0.5% for 30 sec (Sigma, St. Louis, MO). Slides were then dehydrated through 95% alcohol (5 min.) and 2 changes of absolute alcohol, 5 min each, cleared in 2 changes of xylene (5 min) and finally mounted with xylene based mounting medium.

## Results

### Growth of human MPM cells in nude mice

**Figure 1:**
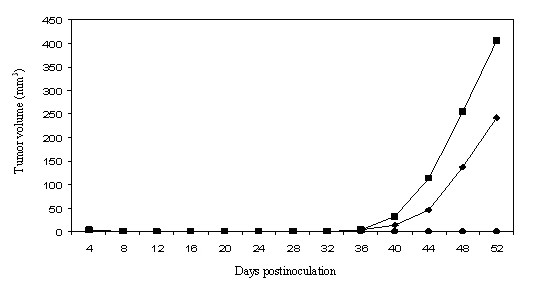
**Subcutaneous growth of MPM cells**. Subcutaneously growth of MPM cells in nude mice. ◆ = IST-Mes3 (2 × 10^6 ^cells injected), ■ = IST-Mes3 (4 × 10^6 ^cells injected), ▲ = IST-Mes2 (2 × 10^6 ^cells injected), ● = MM-B1 (2 × 10^6 ^cells injected). Tumor volume was calculated using the formula: V (mm^3^) = (D × d^2^)/2, where d (mm) and D (mm) are the smallest and largest perpendicular tumor diameters, respectively.

**Figure 2:**
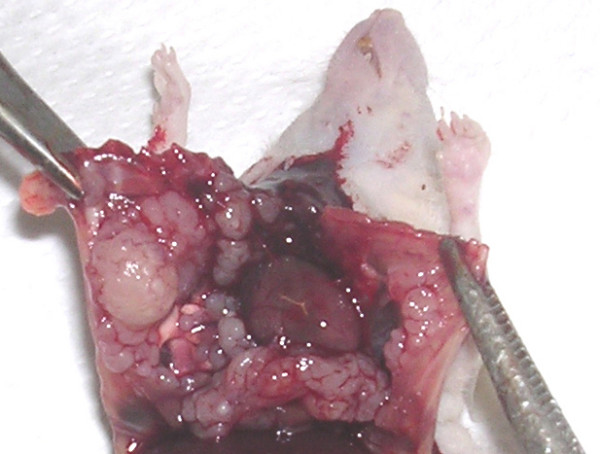
**Pleural MPM nodules**. IST-Mes3 cells (2 × 10^6 ^in 100 μl of HBSS) were injected into the left pleural cavity of nude mice. Tumor masses completely invaded the thoracic and visceral pleural and the lungs. The irregular growth of this cell line is evidenced by a major bulk of tumor in the right chest.

**Figure 3:**
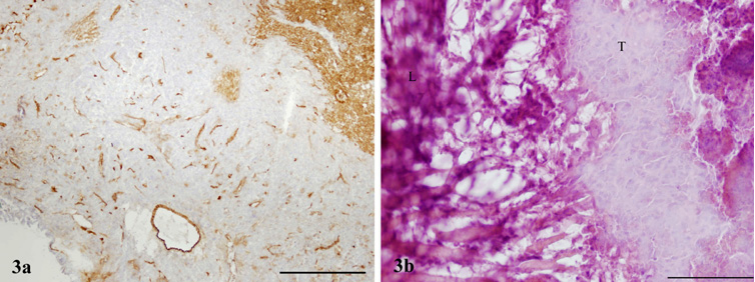
**Orthotopic MPM histology**. Fig. 3a. Frozen sections of tumors grown into the pleural cavity of nude mice injected with IST-Mes3 cells were stained with an anti-mouse CD31 antibody. Tumors were intensively stained revealing an intricate network of microvessels. Bar = 500 μm. Fig. 3b. H&E staining of tumor invading the lung. T: tumor, L: lung. Bar = 100 μm.

Human MPM cell lines were tested for their ability to form tumors after intrapleural and subcutaneous injection in nude mice. Table I summarizes the tumor take rates of these cell lines. Tab. II, and III summarize the growth rate of these cell lines after orthotopic implantation, whereas Fig. 1. shows their growth rate following subcutaneous implantation. IST-Mes2 cells were unable to grow in nude mice. By contrast, 2 × 10^6 ^IST-Mes3 or MMB-1 tumor cells had a take-rate of 100 % when injected into the left pleural cavity. The mean survival time was of 72.5 ± 6.4 days for mice injected with MM-B1 cells; 81.5 ± 21.7 days for mice injected with the IST-Mes3 cells and 69.6 ± 14.5 days for mice injected with the IST-Mes3/2P cells. These mice were sacrificed when moribund, or at different times after cell injection as specified in Tab. I, II, III and IV. Animals injected with the IST-Mes3 cells were analyzed when they became dyspneic because of the tumor growth rate varies from animal to animal. These variations were not observed upon in vitro culture of IST-Mes3 tumor as shown by the analysis of the growth rate of IST-Mes3/2P cells. Tumor cells grew on both sides of parietal and visceral pleura, diaphragm and mediastinum; at later times, tumor invasion into lung parenchyma was observed (Fig. 2, 3). No pneumothorax was evidenced in the mice. Pleural effusions as well as lymph node metastasis were noted only in the later stages of orthotopic growth of MM-B1, IST-Mes3 and IST-Mes3/2P cells. MM-B1 and IST-Mes2 cells did not grow when injected subcutaneously. IST-Mes3 cells started to grow only at forty days after tumor cell injection. This lag phase was independent on the number of cells injected (Fig. 1).

### Immunohistochemical analysis

MPM cells were injected into pleura cavity of nude mice and tumor samples were collected for immunohistochemical analysis. CD31 staining revealed that tumor mass was highly vascularized (Fig. 3). For the phenotypic characterization of tumor mass, a panel of eight markers was used (Tab. 5). Recent findings showed that Mesothelin and TTF-1 have a limited value in assisting in the diagnosis of Mesothelioma and therefore were not used [13,14]. CEA and BerEP4 are adenocarcinoma specific markers and therefore are nor expressed by mesothelioma. Calretinin and HBME-1 are positive markers shared by both epithelioid and biphasic variants of mesothelioma, whereas CD15 is a negative marker. Surface expression of the epithelial membrane antigen (EMA) is a marker that discriminates between reactive proliferation of mesothelial cells and malignant mesothelioma. The epithelia of biphasic mesotheliomas show strong reactivity for Cytokeratin 8/18, whereas Podoplanin is the most recently recognized marker for epithelioid mesotheliomas.

## Discussion

New strategies for MPM treatment, which include inhibition, of angiogenesis, induction of tumor cell apoptosis, gene therapy and vaccines [15], need clinically representative animal models to test new drugs and explore tumor biology.

In this work we established a non invasive orthotopic model of human MPM by injecting the cancer cells directly into the pleural cavity of nude mice. The characteristics of tumor growth resemble those observed in tumor-bearing patients, with colonization of parietal and visceral pleura, diaphragm, mediastinum and, at later stages, lung parenchyma. We analyzed the tumorigenicity of three different human MPM cell lines, which exhibited different biological behaviors in vivo. Interestingly, the growth of MM-B1 cells seems to be site-specific as they did not form tumor masses when injected subcutaneously. Differently, the IST-Mes3 cells grow subcutaneously with a latency which does not depend on the number of cells injected (Fig. 1). This resembles some human situations in which cancer cells start to grow exponentially and form manifest tumor masses only after a long period of time from the acquisition of the tumorigenic phenotype.

As previously reported, neo-angiogenesis is an important process in MPM and protocols to inhibit this process are currently under investigation. Our model can be useful to study angiogenesis in MPM as tumors displayed high density of micro-vessels. IST-Mes3 tumor grew with a latency of 60 days and this period remained substantially constant after tumor passage in mice. All the cell lines tested were unable to form metastases in the visceral organs, likely due to lack of time for tumor dissemination as the rapid spreading of the tumor cells into the pleural cavity dramatically resulted in mouse death. Among the cell lines tested, the MM-B1 cells seem to be the more appropriate for drug evaluation. In fact, as shown in Tab. II, the IST-Mes3 cells grow very rapidly to follow the progression of the disease, colonizing the entire pleural cavity and dramatically affecting the physical conditions of the mice which become cachectic and dyspneic, and the latency of tumor takes varies from animal to animal. The results obtained with the IST-Mes3/2P cell show that these variations are not observed when ex vivo tumor cells are cultured in vitro.

The growth rate of MM-B1 cells is reproducible. Tumors develop in all animals with a similar latency and the rate of tumor growth is slow enough to follow the progression of the disease. In this context, a noninvasive imaging methodology would be a useful tool to follow tumor development.

IST-Mes2 cells did not grow in nude mice, but, since the epithelioid phenotype is the most common sub-type of mesothelioma, we plan to set up an orthotopic model using different ephitelioid cells.

Immunohistochemistry of tumors growing in the nude mice showed that MM-B1 and IST-Mes3 cells maintained the mesothelioma-specific characteristics, thus confirming the validity of the orthotopic model here established.

## Conclusion

Overall, this work describes the biological behavior of human MPM cells injected in the pleural cavity of nude mice. Because of the similarity of this orthotopic model with the human disease, the simplicity of execution and the reproducibility of the results, we propose this model as an useful tool for in vivo MPM studies.

## Competing interests

The author(s) declare that they have no competing interests.

## Authors' contributions

DM performed the orthotopic and subcutaneous implantation of cancer cells in nude mice, the autopsy and the immunoassays. AC carried out the cells culture and participated in the immunohistochemical analysis. GS designed and coordinated the study. PA participated in the coordination of the study and helped to draft the manuscript. SO and RL performed the phenotypic characterization on MPM cells orthotopically implanted in nude mice. All the authors read and approved the final manuscript.

## Pre-publication history

The pre-publication history for this paper can be accessed here:



## Figures and Tables

**Table 1:** Tumorigenicity of MPM cell lines after orthotopic and subcutaneous implantation in nude mice.

Cell line	Histology	Tumorigenicity	Implantation method	Survival(days) (mean ± SD)	Survival (days) (Min/Max)
IST-Mes2	Epithelioid	0/15	Orthotopic	-	-
	Epithelioid	0/15	Subcutaneous	-	-
IST-Mes3	Biphasic	15/15	Orthotopic	81,5 ± 21,7	60/122
	Biphasic	15/15	Subcutaneous	Sacrificed after 90 days	-
IST-Mes3/2P	Biphasic	15/15	Orthotopic	69,6 ± 14,5	54/89
MM-B1	Biphasic	15/15	Orthotopic	72,5 ± 6,4	68/77
	Biphasic	0/15	Subcutaneous	-	-

2 × 10^6 ^MPM cells were injected into the pleural cavity of nude mice. The experiment ended when mice were moribund.

**Table 2:** Growth progression of IST-Mes3 cell line after orthotopic implantation in nude mice.

Days after implantation	<56	60	65	71	73	80	87	94	98
N° of animals with tumor nodules^1^	0/5	1/5	2/5	1/5	1/5	0/5	1/5	1/5	2/5
N° of tumor nodules detected in each animal	-	>20	>20	>20	>20	-	>20	>20	>20

2 × 10^6 ^IST-Mes3 cells were injected into the pleural cavity of nude mice. Five animals were sacrificed when they became dyspneic or at indicated times and tumor nodules were counted, measured, removed and frozen for immunohistochemical analysis. Data shown report tumor development in individual animals.

**Table 3:** Growth progression of IST-Mes3/2P cell line after orthotopic implantation in nude mice.

Days after implantation	54	61	68	75	82	89
Mediastinum nodules	< 10^1^	<10^2^	<10^3^	<10^3^	10–20^3^	10–20^3^
Pericardium nodules	< 10^1^	<10^2^	<10^3^	<10^3^	10–20^3^	10–20^3^
Parietal Pleura nodules	< 10^1^	<10^2^	<10^3^	<10^3^	10–20^3^	10–20^3^
Lungs nodules		-	-	-	<10^2^	<10^2^
Diaphragms nodules	-	-	-	-	<10^2^	<10^2^

2 × 10^6 ^IST-Mes3/2P cells were injected into the pleural cavity of nude mice. At indicated times mice were sacrificed and tumor nodules were counted, measured, removed and frozen for immunohistochemical analysis. Data shown are mean of 5 mice.

^1 ^Nodule diameter ≤ 1 mm; ^2 ^Nodule diameter = 2–3 mm; ^3 ^Nodule diameter ≥ 3. No nodules were found before the days indicated in the table.

**Table 4:** Growth progression of MM-B1 cell line after orthotopic implantation in nude mice.

Days after implantation	38	45	52	59	66	73
Mediastinum nodules	< 10^1^	10–20^1^	10–20^2^	10–20^2^	>20^3^	>20^3^
Pericardium nodules	< 10^1^	10–20^1^	10–20^2^	10–20^2^	>20^3^	>20^3^
Parietal Pleura nodules	-	-	-	10–20^2^	>20^3^	>20^3^
Lungs nodules	-	-	-	10–20^2^	>20^3^	>20^3^
Diaphragms nodules	-	-	-	-	-	-

2 × 10^6 ^MM-B1 cells were injected into the pleural cavity of nude mice. At indicated times, mice were sacrificed and tumor nodules were counted, measured, removed and frozen for immunohistochemical analysis. Data shown are mean of 5 mice.

^1 ^Nodule diameter ≤ 1 mm; ^2 ^Nodule diameter 2–3 mm; ^3 ^Nodule diameter ≥ 3 mm. No nodules were found before the days indicated in the table.

**Table 5:** Phenotypic characterizzation of MM-B1 and IST-Mes3 cells after orthotopic implantation in nude mice.

	**Cell lines**	
**Marker**	**IST-Mes3**	**MM-B1**

CEA	-	-
Calretinin	+	+
CD15 (LeuM1)	-	-
Ber-EP4	-	-
Cytokeratin 8/18	+	+
EMA	+ (membrane)	+ (membrane)
HBME-1	+	+
Podoplanin	+	+

IST-Mes3 and MM-B1 cells were injected into the pleural cavity of nude mice. At indicated times the mice were sacrificed and tumor nodules were removed and frozen for immunohistochemical analysis of mesothelioma-specific markers.
